# Estimating Player Positions from Padel High-Angle Videos: Accuracy Comparison of Recent Computer Vision Methods

**DOI:** 10.3390/s21103368

**Published:** 2021-05-12

**Authors:** Mohammadreza Javadiha, Carlos Andujar, Enrique Lacasa, Angel Ric, Antonio Susin

**Affiliations:** 1 ViRVIG, Universitat Politècnica de Catalunya-BarcelonaTech, Pau Gargallo 14, CS Dept, Edifici U, 08028 Barcelona, Spain; mohammadreza.javadiha@upc.edu; 2 ViRVIG, Universitat Politècnica de Catalunya-BarcelonaTech, Jordi Girona 1-3, CS Dept, Edifici Omega, 08034 Barcelona, Spain; 3 Complex Systems in Sport Research Group, Institut Nacional D’Educacio Fisica de Catalunya (INEFC), University of Lleida (UdL), 25192 Lleida, Spain; elacasa@inefc.es (E.L.); aric@gencat.cat (A.R.); 4 Engineering School (ETSEIB), ViRVIG, Universitat Politècnica de Catalunya-BarcelonaTech, Avda. Diagonal 647, 08028 Barcelona, Spain; toni.susin@upc.edu

**Keywords:** sports science, racket sports, deep learning, pose estimation, player tracking, tracking data

## Abstract

The estimation of player positions is key for performance analysis in sport. In this paper, we focus on image-based, single-angle, player position estimation in padel. Unlike tennis, the primary camera view in professional padel videos follows a de facto standard, consisting of a high-angle shot at about 7.6 m above the court floor. This camera angle reduces the occlusion impact of the mesh that stands over the glass walls, and offers a convenient view for judging the depth of the ball and the player positions and poses. We evaluate and compare the accuracy of state-of-the-art computer vision methods on a large set of images from both amateur videos and publicly available videos from the major international padel circuit. The methods we analyze include object detection, image segmentation and pose estimation techniques, all of them based on deep convolutional neural networks. We report accuracy and average precision with respect to manually-annotated video frames. The best results are obtained by top-down pose estimation methods, which offer a detection rate of 99.8% and a RMSE below 5 and 12 cm for horizontal/vertical court-space coordinates (deviations from predicted and ground-truth player positions). These results demonstrate the suitability of pose estimation methods based on deep convolutional neural networks for estimating player positions from single-angle padel videos. Immediate applications of this work include the player and team analysis of the large collection of publicly available videos from international circuits, as well as an inexpensive method to get player positional data in amateur padel clubs.

## 1. Introduction

In the last few years there has been an increasing interest in player tracking techniques [1] as well as in video analysis for sports [2]. Recent advances in computer vision techniques, especially in object detection, segmentation, and pose estimation methods, have opened new opportunities for image-based performance and tactical and biomechanical analyses in sport. In this paper we focus on player positions in padel, a modern racquet sport born in the 1970s with a relevant growth in the number of players [3]. The dimensions of the court (smaller than tennis ones) and the presence of walls surrounding the court, which facilitate returning the ball, allow padel to be practiced by people of any physical condition [3].

The use of computer vision techniques in related racquet sports (such as tennis) has been extensively studied for tasks such as player tracking [4,5,6], ball tracking [7,8,9,10], content-based retrieval [11,12], virtual replays [7], and automatic annotation [13].

However, unlike major racquet sports, padel matches exhibit distinctive features which hinder major computer vision tasks such as player tracking. It is almost exclusively played in doubles, which increases the risk of interplayer occlusion in low-angle and moderately high-angle shots, and the playing field is enclosed by walls: glass walls might show reflected people from the public, side glass walls might reflect the players themselves, metal mesh panels over the glass walls partially occlude a part of the field, and other structural elements connecting the glass panels also occlude some parts of the court.

**Figure 1:**
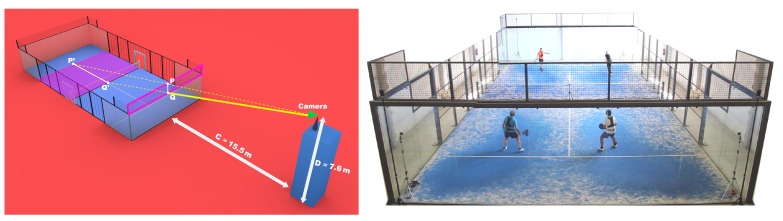
Optimal camera placement for padel: if the main camera (shown in green) is placed at the intersection of lines $PP^{\prime }$ and $QQ^{\prime }$, the mesh over the front wall (highlighted in pink) occludes the portion of the court between the net and the opposite service line. Notice that, for this camera position, the segment PQ (on the mesh) and the segment $P^{\prime }Q^{\prime }$ (on the court) overlap in camera space. Another option, requiring less physical space around the court, is to place the camera on the straight line defined by *Q* and $Q^{\prime }$ (on the portion shown with a thick yellow line), as this ensures that the closest half of the court will not be occluded by the mesh. Right: View of an amateur padel court from a camera close to the de facto standard for padel streaming. In this example, the mesh roughly occludes the upper half of the court.

The occlusion of the mesh panel and structural elements is particularly important, and the de facto camera angle for padel broadcasting is chosen to minimize the impact of such occlusion. In the standard setting (Figure 1), the image-space projection of the mesh panel spans the region from the bottom part of the net to near the opposite service line, which is achieved by placing the camera at about 7.6 m from the floor, and 15.5 m from the glass panels. We have observed that most professional padel videos are recorded following this camera angle.

Therefore, the analysis of padel videos exhibits unique features due to the presence of walls surrounding the court and their impact on visibility occlusion and game development. A few papers do analyze player position and displacement aspects in padel but most of them are based on data from direct observation [14] or video analysis from zenithal [15,16] or nearly zenithal cameras [17], which greatly simplify player detection.

To the best of our knowledge, no formal analysis has been made about the accuracy of video analysis techniques for player tracking on padel videos recorded from nonzenithal cameras. The study closest to this work refers to video analysis for squash [18] using a human pose estimation algorithm [19]. The camera angle for the videos in [18] is close to those in major padel circuits. However, we analyze many state-of-the-art computer vision algorithms besides [19] in a more challenging scenario due to the occlusion of the multiple elements in a padel court (Figure 1).

In this paper we analyze the performance and accuracy of state-of-the-art position estimation methods on a large collection of frames selected from publicly available videos. Our aim is to estimate the 2D position of players within the court. Such 2D positions can be estimated from the player’s 2D bounding boxes, from their segmentation masks, or from player keypoints (e.g., feet and hip). Therefore, object detection techniques (providing bounding boxes of detected instances), image segmentation techniques (providing pixel-wise masks), and pose estimation techniques (providing keypoints according to some human skeleton model) are good candidates for estimating 2D player positions.

*Detection* algorithms can be trained to recognize and locate instances of multiple objects in an image. A typical output of these algorithms is a collection of bounding boxes enclosing the detected instances [20,21,22,23,24].

*Image segmentation* algorithms (e.g., refs. [23,25,26]) label each pixel of the image with the ID of the predicted instance class corresponding to the pixel. Resulting masks provide more information about the detected objects than just an enclosing box.

*Pose estimation* methods estimate the location of keypoints (e.g., neck, hip, feet) of the detected people. Current human pose estimation methods can be divided into top-down and bottom-up methods. *Top-down* methods (e.g., refs. [21,27,28,29]) first detect person instances, and then their individual joints. An external detector locates person instances and outputs estimated bounding boxes; the top-down method then performs single-person pose detection on the cropped and rescaled subimage. Top-down methods are less sensitive to the scale variance of persons but are usually slower and their performance is constrained by that of the external detector. *Bottom-up* methods (e.g., refs. [30,31]) detect first all keypoints in the image, and then these keypoints are grouped into person instances. Since these approaches operate end-to-end, they are usually faster (even real-time [19]), but most bottom-up methods are quite sensitive to scale variation.

We selected state-of-the-art methods for object detection, refs. [20,22,23,32] segmentation [23,24,25], and pose estimation, [19,21,29,30,31] all of them based on deep convolutional neural networks. For the sake of reproducibility, all techniques we tested come from publicly available computer vision repositories including neural network architectures, pipeline configurations, and trained models.

For evaluating the accuracy of these methods, we used a collection of frames extracted from 24 videos from World Padel Tour (WPT), the major padel international circuit. We provide links to these publicly available videos in Table S1 of the Supplemental Material. In order to evaluate the accuracy of the selected techniques, ground truth positions were obtained by annotating manually player keypoints (left/right ankles and hip). These three keypoints allowed us to estimate an image-space player position, which was then converted onto a court-space position. We then computed deviations from predicted and ground-truth player positions.

## 2. Materials and Methods

### 2.1. Dataset Description

Our evaluation is based on frames selected from professional padel videos. In particular, we selected 24 matches from World Pader Tour, all of them publicly available on the WPT YouTube channel (see links in Table S1 of the Supplemental Material). All images were analyzed at 1920 × 1080. In all videos the primary camera roughly follows the standard view (Figure 1). The selected set is varied in terms of gender (15 male, 9 female finals) and lighting conditions (16 indoor, 8 outdoor).

We selected randomly two game points for each match (24 × 2 = 48 periods), with the only requirements that the whole period was captured from the main camera and that all four players were inside the court during the whole period.

For the first dataset (test1), we selected a 5-image sequence from each game point (48 × 5 = 240 frames). Selected frames within each sequence were 10 frames apart (i.e., 400 ms step for 25 fps videos). These frames were manually annotated by 5 annotators who identified keypoints of the four players, as described in Section 2.2.

For the second dataset (test2), we selected also the in-between frames (using consecutive frames, i.e., 40 ms apart for 25 fps videos) from each game point (48 × 40 = 1920 frames). These frames were not annotated but relatively close (at most 400 ms) to a pair of annotated images from test1.

### 2.2. Defining 2D Court-Space Positions

Given a frame, we are interested in estimating the 2D position of the players within the court. Such 2D position $P=(P_{x},P_{y})$ must be represented in court-space (using physical length units e.g., meters) to enable further sport analyses based on positions and displacements. The estimation of the vertical displacement of the player with respect to the court floor is out of the scope of this paper.

**Figure 2:**
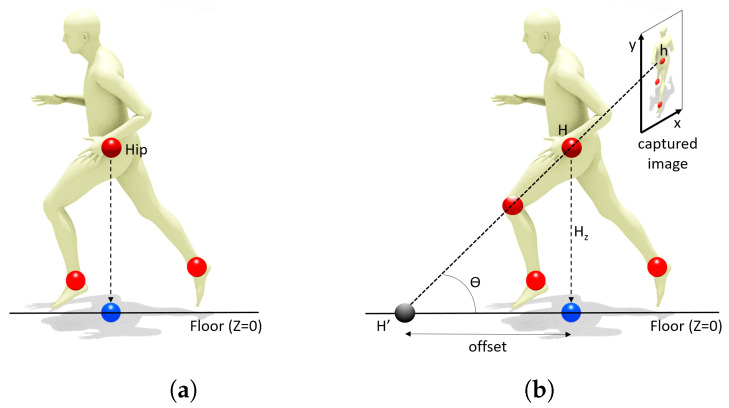
Our definition of 2D position of the player (**a**) is conceptualized as the vertical projection of the hip joint onto the floor (blue sphere). The perspective transform used to convert image points (e.g., hip) to court-space points (**b**) creates an offset that depends on the distance to the floor and the camera angle.

Since players move in a 3D world, one way of formalizing such 2D position is considering the vertical projection of some point near the player’s center of mass. Although the center of mass of a human body depends both on anatomical and pose factors, a good estimate is the center of the hip bones. The hip is actually a major keypoint in most human models used in computer animation and computer vision, and thus a common output for pose estimation methods. Figure 2a shows our conceptualization of the 2D position of a player (blue sphere), as the vertical projection of the hip (red sphere).

### 2.3. Court-Space Positions from Image-Space Joints

Since the images we are interested in are captured by a conventional camera, we need to convert image-space data (player bounding boxes, segmentation masks, pose keypoints) to court-space positions. Should images come from a depth camera, a zenithal camera or multiview cameras, such conversion would be robust and straightforward. In our case though, we need to resort to a 4-point perspective transform, which will allow us to perform such conversion for player parts *on the floor*, or just a little bit off the floor (e.g., feet).

We observed that spherical distortion was negligible in all selected videos, so we performed no image undistortion. For each video, we obtained the pixel coordinates of the quadrangle defined by the four corners of the court. We then computed the 3 × 3 matrix of the perspective transform that maps this source quadrangle (in homogeneous *image-space coordinates*) onto the 10 m × 20 m destination quadrangle corresponding to the physical size of a padel court (*court-space coordinates*). We use this matrix to map points from image-space to court-space, but this is only exact for body parts *on the floor*. Figure 2b illustrates this problem. Multiple body parts (e.g., the hip and the left knee) are projected onto the same image-space pixel *h*. Since no reliable depth estimation is available, applying the perspective transform to *h* leads to an offset between the estimated position (black sphere) and the true one (blue sphere). The offset can be computed as $\frac{H_{z}}{\tan(\theta )}$, being thus 0 when either the body part is on the floor ($H_{z}=0$) or the camera angle θ equals 90 (zenithal camera).

**Figure 3:**
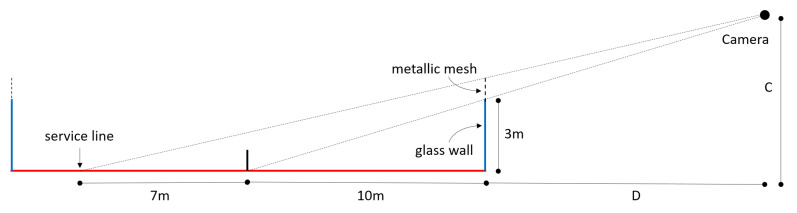
Side view of a padel court showing placement for the camera to satisfy the occlusion constraints.

Since the offset is inversely proportional to tan(θ), we computed the interval of θ values for the de facto stardard camera view. First, we computed the location of the camera in that setting. The constraint that the image-space projection of the mesh spans the range from the net to the service line leads to the following system (Figure 3):

$$\left\{\begin{array}{c} \frac{C}{D+10}=\frac{3}{10}\\ \frac{C}{D+17}=\frac{4}{17} \end{array}\right.$$

The solution $C=\frac{84}{11}$, $D=\frac{170}{11}$ indicates that the camera should be placed ≈7.6 m above the court, and ≈15.5 m from the wall (see Figure 1).

Within the court, θ ranges from $\arctan\frac{C}{D}\approx 26.3{}^{\circ}$ at the bottom edge of the court, to $\arctan\frac{C}{D+20}\approx 12.5$ at the top edge. The ratios $\frac{D}{C}$, $\frac{D+10}{C}$ and $\frac{D+20}{C}$ can be interpreted also as the error amplification factors at different parts of the court. For example, if the feet of the player is (or is estimated at) some height *c* above the floor, the approximate offset (error) in the vertical *Y* coordinate of its estimated image 2D position will be 2.0c, 3.3c or 4.64c, depending on whether the player is located near the bottom edge, the net, or the top edge of the court. Therefore, we expect position predictions to be less accurate for players on the top half of the court, since their screen-space projection is smaller (thus hindering detection, segmentation, and pose estimation tasks), their occlusion higher, and because vertical errors will have a larger amplification factor.

Notice that the above amplification factors apply both to manual and automatically computed annotations.

### 2.4. Manual Player Annotation

**Figure 4:**
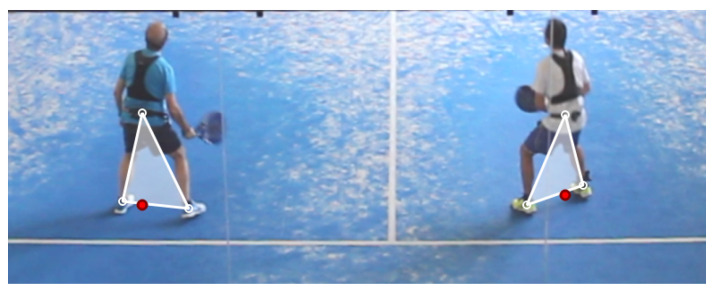
Close-up view of the image annotation application showing manually annotated joints (white circles) and estimated image-space player positions (red circles).

Since no ground truth positions are available for elite padel videos, we created a simple application for their manual annotation. In a pilot study, we observed that directly estimating the 2D position (as the vertical projection of the hip) was a difficult task. Therefore, we decided to ask the annotators to identify three joints for each player: the left ankle $\left(L_{x},L_{y}\right)$, the right ankle $\left(R_{x},R_{y}\right)$, and the hip center $\left(H_{x},H_{y}\right)$ (Figure 4). From the image-space positions of these joints, we estimated the image-space position of the player as $p=(p_{x},p_{y})$ with $p_{x}=H_{x}$ and $p_{y}=\left(L_{y}+R_{y}\right)/2$ (Figure 4). That is, we use the hip to estimate the image-space horizontal coordinate and the average of the feet to estimate the vertical coordinate (near the bottom part of the image-space projection of the player). The court-space position was computed by multiplying the perspective transform matrix.

**Figure 5:**
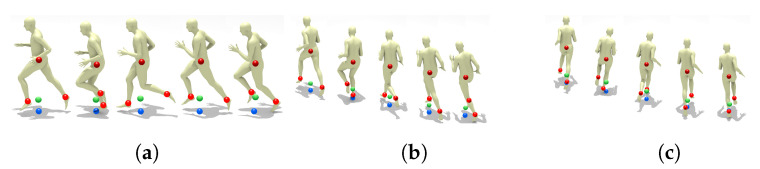
Deviation between the true image-space 2D position (blue spheres) and the position estimated from the feet and hip joints (green spheres), for different poses and orientations: (**a**) side view, (**b**) three-quarter view, and (**c**) back view. Notice that the vertical displacement (which will be amplified due to the perspective transform) is small and roughly constant along the running animation cycle. Motion data from subject #9, trial 6 of CMU Motion Capture Database.

For players standing or walking, the method above provides an accurate estimate of the true court-space 2D position. For running players though we expect a larger error, since at some frames both feet are in the air. We analyzed some running cycles by rendering a realistic human avatar [33] using Motion Capture Data from Carnegie Mellon University Motion Capture Database http://mocap.cs.cmu.edu/, accessed on 1 March 2021. Figure 5 shows such deviations (in image-space) for different player poses and orientations. Fortunately, the vertical displacement is small and roughly constant along running animation cycles.

For human annotators, obtaining accurate labels for a large number of examples is often infeasible. In our case, the position of player keypoints (ankles, hip center) must be guessed since the joints are not directly visible and occluded by skin and clothes. Video compression artifacts, motion blur, and occluding objects further hinder this task. Collected positions are thus subjective and noisy. We thus asked M = 5 participants to annotate all images in test1. For each image and player, court-space positions estimated by the 5 annotators were aggregated to estimate a ground truth position for each of the $N_{1}$ = 960 observations. Following Raykar et al. [34] we estimate ground truth positions for each player by computing a weighted average of the annotated positions. Annotator weights are initialized as uniform weights. Then the aggregated position is recomputed using the current weights, which are then updated as the inverse variance with respect to the updated average. This process is repeated until convergence (usually less than 10 iterations). The aggregated position is taken as the ground truth 2D position for test1.

**Figure 6:**
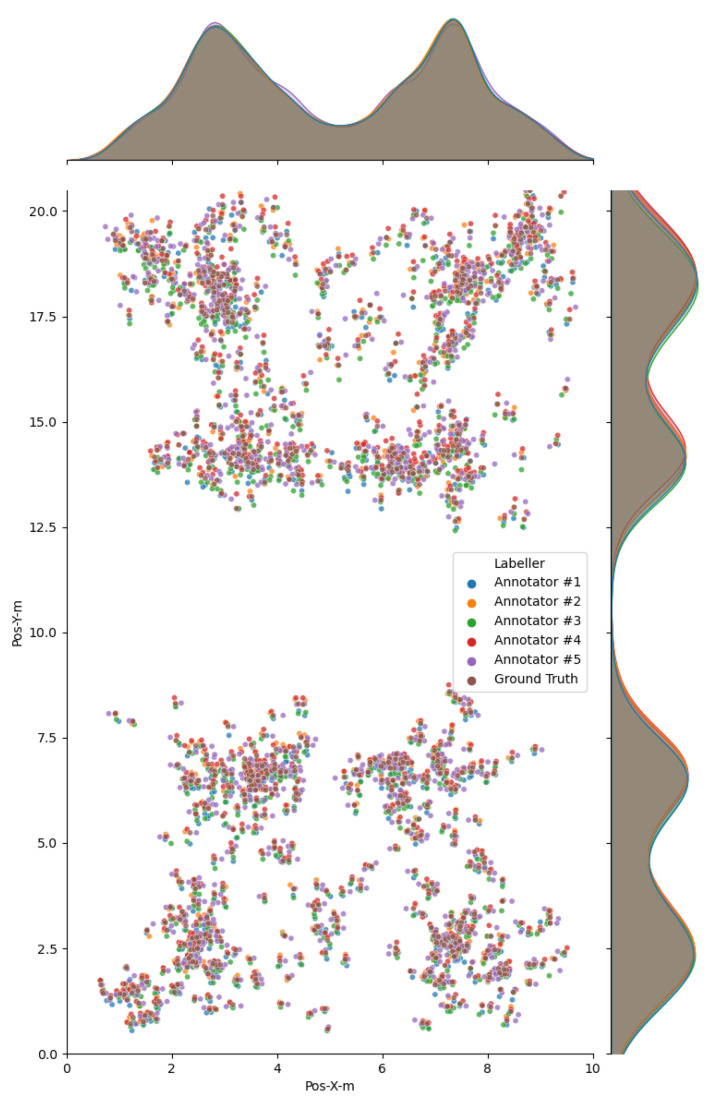
Estimated court-space player positions for all annotators. Top and right: Gaussian kernel density estimates.

Figure 6 shows the estimated positions before and after aggregation. Notice that per-annotator estimates are grouped into tight clusters around the estimated ground truth. We computed the error of per-annotator positions with respect to ground truth positions, as a measure of the difficulty of the annotation task as well as a measure of consistency of the estimated ground truth. As expected, the mean error was negligible (0.22 cm and −0.97 cm for X and Y coordinates) since the aggregation method we used is quite robust to outlier annotations. The standard deviation (SD) was 5.92 cm and 12.15 cm for X, Y coordinates, which better reflect the overall accuracy of human annotators in this particular task. Since the error refers to court-space positions, this already accounts for the error amplification due to the perspective deformation, which mostly affects Y coordinate.

### 2.5. Selected Position Estimation Methods

We tested both top-down and bottom-up pose estimation methods. Top-down methods use an external person detector to locate person instances, so that subsequent pose estimations are limited to a single-person within each detected bounding box. Table 1 lists the state-of-the-art top-down pipelines we selected for evaluation. All pipelines belong to the OpenMMLab Pose Estimation Toolbox available at https://github.com/open-mmlab/mmpose, accessed on 1 February 2021. Notice that each pipeline is based on multiple methods (e.g., the backbone for extracting feature maps and the keypoint head for pose estimation).

On the other hand, bottom-up methods first locate keypoints for all the persons in the image, and then merge them into person instances. Table 2 lists the state-of-the-art bottom-up pipelines we selected for evaluation. All pipelines belong to the OpenMMLab Pose Estimation Toolbox except OpenPose [19].

Regardless of the pose detection method, the image-space 2D position was computed from hip/ankle coordinates as $p=(H_{x},\frac{1}{2}\left(L_{y}+R_{y}\right))$. Since we chose the COCO human model for all tests, the hip itself was computed as the average of the left and right hip keypoints.

We also evaluated the accuracy of the detectors that were selected for the person detection step of top-down pose estimation methods. All these detector pipelines are part of the OpenMMLab Detection Toolbox [20] available at https://github.com/open-mmlab/mmdetection, accessed on 1 February 2021. In this case, the estimated 2D position was just the mid point of the bottom edge of the box.

**Figure 7:**
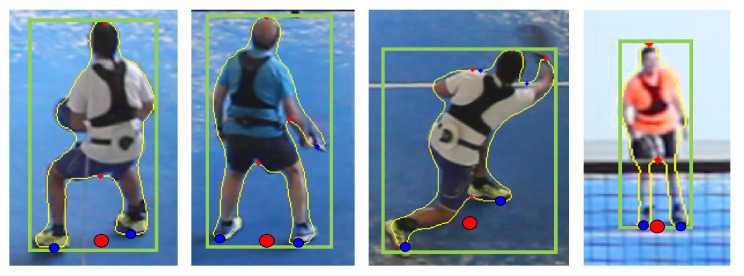
Segmentation-based estimation: mask contours (in yellow), mask bounding boxes (in green), local minima in the vertical direction (blue dots), points used to estimate feet locations (blue circles), and estimated player positions (red circles).

Finally, we also report the accuracy of segmentation methods. We used Detectron2 [24], available at https://github.com/facebookresearch/detectron2, accessed on 1 February 2021. We extracted the contour of detected person instances and estimated player positions by combining feet and hip estimates. We used contour points at local minima (bottom part of the instances) to estimate image-space feet locations (Figure 7) and the minimum of all detected local maxima to estimate the hip.

## 3. Results

### 3.1. Test 1

We tested all the methods in Table 1 and Table 2 with the images in test1, all of them from professional padel videos (see Table S1 of the Supplemental Material).

Since annotated-based ground-truth data was available for test1, we could compute different accuracy and precision metrics. For each of the $N_{1}=960$ court-space position estimations, the error was computed separately for X and Y coordinates by subtracting the ground truth. For each method, we report the absolute mean error $\bar{|E_{x}|}$, $\bar{|E_{y}|}$, as well as the standard deviation of the error $s(E_{x})$, $s(E_{y})$. Some methods were unable to detect all four players; the percentage of detected players ranged from 86% to 99.8%, depending on the method. Since we ignored missing detections when computing $\bar{|E_{x}|}$, $\bar{|E_{y}|}$, $s(E_{x})$, $s(E_{y})$, we also computed the Average Precision (AP) of each method for different distance thresholds. For example, AP${}_{x}^{10}$ is the ratio of correct positions (in this case, with an absolute error below 10 cm) over the total number of observations $N_{1}$. Notice that missing detections do decrease the AP but have no impact on $\bar{|E_{x}|}$, $\bar{|E_{y}|}$, $s(E_{x})$, $s(E_{y})$.

Table 3 summarizes the results of each method, sorted by AP${}_{y}^{50}$ (last column). The prefix of each method indicates its type: DT = detection, MK = mask-based, BU = bottom-up pose detection, TD = top-down pose detection.

**Figure 8:**
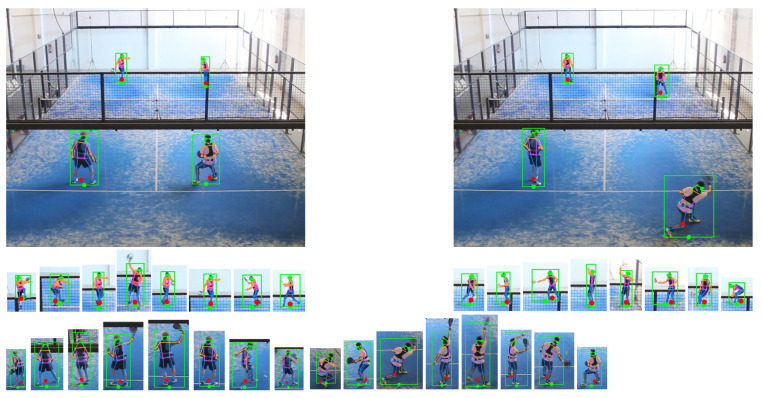
Estimated 2D positions from pose estimation (red circles) and detection methods (green circles). The close-up images show detected boxes and joints for a varied set of game situations, for all four players.

We first discuss metrics on the X coordinate. The mean absolute error was below 3 cm for most methods, which is negligible in a court 10 m wide. The standard deviation, which accounts for random errors, better represents the accuracy of the methods. Most methods achieved $s(E_{x})$ values below 5 cm. Detection methods (providing only bounding boxes, Figure 8) were clearly more inaccurate than pose estimation methods. We computed the bias-corrected Average Precision for 10 cm and 20 cm thresholds, AP${}_{x}^{10}$ and AP${}_{x}^{20}$. Best methods (top-down pose estimators) achieved AP${}_{x}^{10}>98\%$, which is an excellent result.

We now discuss the Y coordinate. Recall that predicting court-space 2D positions is harder for this coordinate due to higher mesh/structure occlusion, smaller player detections in image-space, and higher error amplification for misplaced keypoints. The mean absolute error was below 12 cm for the best methods, which also achieved $s(E_{y})$ values below 11 cm. We computed the bias-corrected Average Precision for 30 cm, 40 cm, and 50 cm thresholds, AP${}_{y}^{30}$, AP${}_{y}^{40}$, and AP${}_{y}^{50}$. Again, the best methods were top-down pose estimators, which achieved AP${}_{y}^{40}>95\%$ and AP${}_{y}^{50}>98\%$. If we take AP${}_{y}^{50}$ as a global measure of the method’s precision, the best method was the combination of Faster R-CNN [22] for player detection, with HRNet [30,31] for locating the keypoints of each detected player.

Overall, nonsystematic errors for predicted court-space 2D positions were quite reasonable for the best computer vision methods. Actually, in our comparison, computer vision methods offered approximately the same accuracy as human annotators. Recall that the standard deviation for human annotators (Section 2.4) was 5.92 cm for X coordinates (vs. approx. 5 cm for the best methods in Table 3), and 12.15 cm for Y coordinates (vs. approx. 12 cm).

We also computed systematic and random errors for players on the bottom part of the court, which are nearer to the camera and thus easier to locate. As shown in Table 4, the results are significantly better, with random errors around 3 cm and 7 cm for X and Y coordinates, respectively and more than 98% of the estimations below the 30 cm threshold for the best methods. Again, if we take AP${}_{y}^{50}$ as a global measure of the method’s precision, the best method for the players on the bottom part of the court was again HRNet [30,31], used in combination with either a Faster R-CNN detector [22] or Hybrid Task Cascade detector [25]. Notice also that for these players, some bottom-up pose estimation methods provide more competitive results, which confirms the difficulty of bottom-up methods to estimate joints in persons with small image-space projections.

We also tested the best detection/pose estimator pipelines on a more challenging image set from an amateur video, for visual evaluation. All players were informed and gave their consent on this study. Figure 8 shows the estimated image-based positions for a representative collection of frames, with different types of shots, poses, displacements, and location within the court. Notice that hip and ankle joints are detected very robustly, even in challenging poses with partial occlusions due to the metal structure and the mesh over the glass walls (in elite padel videos these metallic parts are thinner and less obtrusive; see sample video in the supplemental material. Estimates from the ankle/hip joints (red circles) provide a good approximation of our conceptualization of 2D player positions. Detection-based estimates are faster but much less accurate (green circles) and sensitive to limb locations.

### 3.2. Test 2

According to Table 4, one of the most accurate methods is the top-down pose estimation configuration that combines a Hybrid Task Cascade detector [25] (htc x101 64 × 4d) based on ResNeXt [36] with a HRNet [30] pose estimator (hrnet w48 256 × 192). We tested the performance of this configuration on a larger dataset (test2) with $N_{2}$ = 7680 observations. For test2 we had ground truth positions only for one every ten frames. We also computed Average Precision values, but this time the predicted court-space position was considered correct if X and Y coordinates were within the intervals defined by the ground truth values at the two nearest frames from test1 (at most 200 ms apart from the frame being analyzed). For example, if ground-truth (x,y) positions for frames *i*, i+10 were $\left(x_{i},y_{i}\right)$ and $\left(x_{i+10},y_{i+10}\right)$, then for a frame *j* with i<j<i+10 a prediction $\left(x_{j},y_{j}\right)$ is considered correct if $x_{j}\in \left[x_{i},x_{i+10}\right]$ and $y_{j}\in \left[y_{i},y_{i+10}\right]$, since the linear interpolation of $\left(x_{i},y_{i}\right)$ and $\left(x_{i+10},y_{i+10}\right)$ would (wrongly) assume constant speed within the 400 ms interval that separates ground truth frames.

We considered thresholds of 30 cm, 40 cm, and 50 cm. For the best configuration above, AP${}^{40}$ was 98.27% and AP${}^{50}$ was 99.17%. When considering only players on the bottom half of the court, AP${}^{30}$ was 99.71%, AP${}^{40}$ was above 99.99% and AP${}^{50}$ was 100%. These results demonstrate that pose estimation methods provide a robust way to estimate player positions and that these positions are indeed very accurate for the players on the bottom half of the court.

### 3.3. Amateur Video

**Figure 9:**
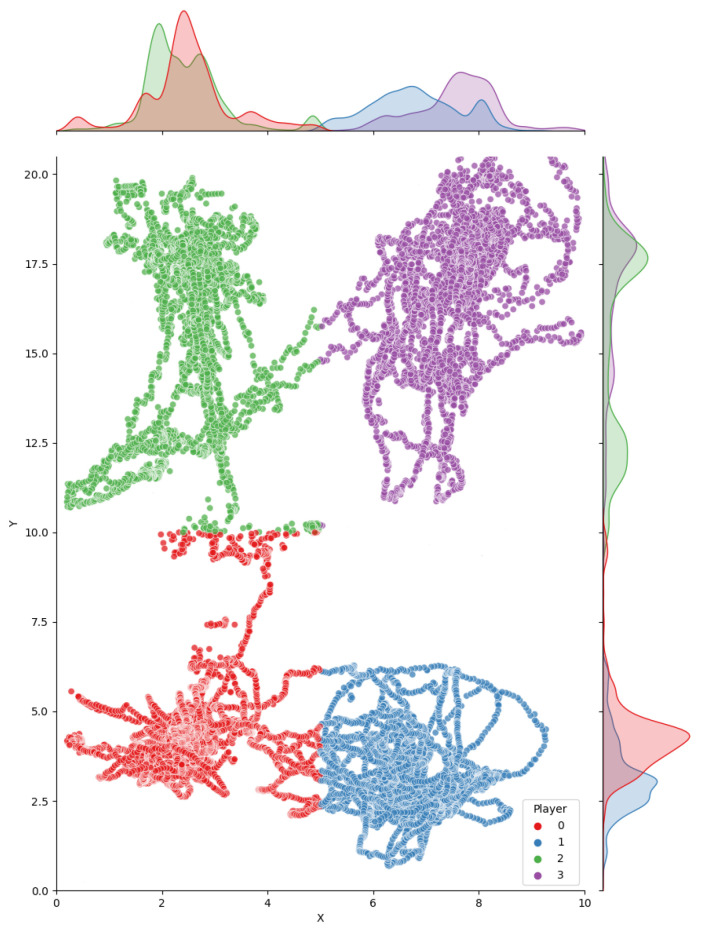
Estimated court-space positions from the accompanying amateur video. Notice that zigzagging is more noticeable for paths on the top part of the court. Hue just indicates the court quadrant the player is located at.

We also tested the best method on a more challenging dataset. The accompanying video shows estimated player positions for a Full HD amateur video. This video is particularly challenging due to the lighting conditions and the thick metallic parts that cause significant occlusion. Furthermore, the camera was closer to the ground than the de facto standard (about 4.6 m vs. 7.6 m), which increased significantly the error amplification for players on the top half of the court. Unlike the videos we used for test1 and test2, the amateur video did show some spherical distortion that we did not correct, and court limits were not clearly visible due to occlusions, which hindered the identification of the court corners needed to compute the perspective transform matrix. Despite all these challenges, even raw (i.e., unfiltered) estimated court-space positions match reasonably well the actual player positions, for the vast majority of frames (see Figure 9 and the accompanying video).

## 4. Discussion

Although a number of technologies have been proposed to get motion data in racket sports, including 3D optical systems based on retroflective markers captured by multiple cameras [38], and Inertial Measurement Units (IMUs) [39,40,41], these approaches require players to wear the IMUs/markers and thus cannot be applied to analyze already existing videos. The marker-less, video-based techniques we compared in this paper fill this gap by allowing the analysis of elite and amateur videos captured in noncontrolled setups.

Overall, the main issues that hinder video-based positional analysis in padel videos are occlusion (net, metallic structure, glass walls, other players) and the error amplification due to the chosen camera angle (Figure 1). This contrasts with the much easier setup of tracking players from a zenithal camera, which does not suffer from occlusion and perspective problems. We have shown that state-of-the-art detection and pose estimation methods do handle occlusion very well.

The simplest approach is to use a person detector network to estimate a bounding box for each player. This approach provides a reasonable localization of the player in the image (methods prefixed with *DT* in Table 3), with random errors around 18 cm for the Y coordinate. Pose estimation methods offer more accurate results, since they identify player joints (hip, feet) that lead to more accurate player positions. Within this category, bottom-up pose estimation methods (prefixed with *BU* in Table 3) are faster than top-down methods (prefixed with *TD* in Table 3) but resulting random errors are above 13 cm for the Y coordinate, whereas most top-down methods achieved errors below 11 cm. As shown in Figure 8 and the accompanying video, even completely occluded arms and legs are estimated at plausible locations.

Since the main camera is static, a simple baseline approach for player detection would be background subtraction [6,16]. For nonzenithal views though, this baseline is less robust to occlusions than the deep segmentation approaches we tested, which in turn were clearly outperformed by pose estimation methods (Table 3 and Table 4), which benefit from keypoint locations within the player’s pixels to predict player positions more accurately.

Notice also that the best methods detected all four players in all or nearly all frames, without requiring video-based object detection techniques [42,43,44] which exploit temporal coherence across consecutive frames. We did not apply any temporal filtering to the data, as this would partially hide the actual accuracy of the methods being compared. For player and team analyses that require the identification (besides localization) of individual players across frames, person detectors can be combined with temporal tracking techniques [45,46].

It must be noted that the results in Section 3 are based on comparing predicted court-space positions with ground truth positions extracted from human-annotated data. Since the perspective correction assumes that the player’s feet are on the ground (height ≈ 0), we have not considered the error for players with both feet in the air, for example during a smash jump. For the standard camera setting, such vertical displacements result in an offset on the predicted court-space Y coordinate. We believe though that these displacements should have a negligible impact on tactical analyses of padel matches. On one hand, jump frequency in padel is relatively small. According to [3], the frequency of *smash jumps* was found to be below 0.6 jumps per minute (professional male players), which represents about 0.0001% of the frames (assuming a vertical jump height of *d* = 1.25 m and duration $t=2\sqrt{2d/g}=1$ s). Split-steps (small vertical jumps used as a preparatory motion for a lateral displacement [47]) were more frequent (1.5 per minute), but since their vertical distance is small, their impact on accuracy is also small.

On the application side, the systematic collection of player tracking data will allow analysts to apply to padel some studies that have been introduced recently in other sports. Some example analyses that only require player positional data include (a) the frequency at which players stay in particular court regions (e.g., attack, defense and transition zones) and how this frequency varies depending on game, player, gender, and skill factors, (b) the detection of time periods where player locations are nonoptimal, e.g., leaving large portions of the court out-of-reach, (c) the quality at which teammates move synchronously within the court, and (d) the distance covered by the players, speed profiles, and sudden direction changes, for the different court zones and game situations. If combined with player *identification* (besides localization), these techniques will allow for further studies such as players’ external load quantification [48], individual action recognition [49], and interpersonal coordination evaluation [50]. As an inexpensive method to get player tracking data, these techniques will allow amateur padel clubs to evaluate and monitor players’ performance, as well as other applications such as video-based reflective learning [40], the analysis of the effects of different training drills, and the comparison between small game adaptations for children.

## 5. Conclusions

In this paper we have compared state-of-the-art position estimation techniques (based on detection, segmentation, and pose estimation) on the high-angle videos that are the de facto standard in padel matches. The best results were obtained by top-down pose estimation techniques, in particular when combining cascade detectors [25] based on ResNeXt [36] with a HRNet [30] keypoint estimator. These methods achieved standard deviations (random errors) below 5 cm (X coord) and 12 cm (Y coord), which are on par with those from human annotators. Average precision values demonstrate the robustness of these methods, with more than 98% of the estimated positions within a 30 cm error tolerance with respect to ground truth, for players on the bottom half of the court. These error values are quite competitive compared to state-of-the-art systems based on multiple-cameras, radar-based positioning, and GPS equipment used in other sports [51].

We chose to estimate player positions using a simple combination of hip and ankle coordinates. Should appropriate training examples be available, we could train a network to predict positions from multiple joints, taking into account also the perspective transform. Actually, as future work we plan to train such a network using realistic human models [33]. We also plan to apply skeleton-based action recognition techniques [52] to detect actions (e.g., smash jumps) that imply a vertical displacement of the player above the ground.

## Figures and Tables

**Table 1:** Top-down pose estimation methods selected for evaluation. The first column shows the detector configuration, i.e., the method used to detect players in the image. The second column shows the actual pose estimator, which computes the keypoints of each player within the bounding box returned by the detector. The following abbreviations are used in the first column: *r50* refers to the use of ResNet-50 as backbone for the detector, *fpn* refers to the use of ResNet plus a Feature Pyramid Network for object detection, *person* indicates the network has been trained on a COCO subset (with person class only), *x101* refers to the use of ResNeXt-101 as backbone for the detector, 32 × 4d and 64 × 4d refer to the ResNeXt-101 template, and *htc* refers to Hybrid Task Cascade [25]. The following abbreviations are used in the second column: *hrnet w48* refers to the HRNet network proposed in [30]; w48 represents the width of HRNet subnetworks, 384 × 288 and 256 × 192 refer to the image resolution for pose estimation (for each detected player), and *dark* refers to DarkPose [29].

Detector Config	Pose Estimator Config	Core Method(s)
faster rcnn r50 fpn person	hrnet w48 384 × 288	HRNet [30] Faster R-CNN [22] ResNet [35]
faster rcnn x101 64 × 4d fpn	hrnet w48 384 × 288	HRNet [30] Faster R-CNN [22] ResNeXt [36]
faster rcnn r50 fpn	hrnet w48 384 × 288 dark	HRNet [30] DarkPose [29] Faster R-CNN [22] ResNet [35]
faster rcnn r50 fpn	hourglass52 384 × 384	Hourglass [27] Faster R-CNN [22] ResNet [35]
faster rcnn r50 fpn	hrnet w48 384 × 288	HRNet [30] Faster R-CNN [22] ResNet [35]
cascade mask rcnn x101 64 × 4d fpn	hrnet w48 256 × 192	HRNet [30] ResNeXt [36] Cascade Mask R-CNN [23]
htc x101 64 × 4d fpn	hrnet w48 256 × 192	HRNet [30] ResNeXt [36] Hybrid Task Cascade [25]
dcn/cascade mask rcnn x101 32 × 4d fpn	hrnet w48 256 × 192	HRNet [30] ResNeXt [36] Cascade Mask R-CNN [23] Deformable ConvNets [37]

**Table 2:** Bottom-up pose estimation methods selected for evaluation. The following abbreviations are used in the first column: *hrnet* and *higher hrnet* refer to the networks proposed in [30,31], 32 and 48 represent the width of HRNet subnetworks, 512 × 512 and 640 × 640 refer to the downsampled image resolution for pose estimation, *res50*, *res101* and *res152* refer to the type of ResNet network [21], and *udp* stands for Unbiased Data Processing for Human Pose Estimation [28].

Configuration	Core Method(s)
higherhrnet/higher hrnet32 512 × 512	Higher HRNet [31]
higherhrnet/higher hrnet32 640 × 640	Higher HRNet [31]
higherhrnet/higher hrnet48 512 × 512	Higher HRNet [31]
hrnet w32 512 × 512	HRNet [30]
hrnet w48 512 × 512	HRNet [30]
mobilenetv2 512 × 512	MobileNetv2 [26]
resnet/res50 512 × 512	ResNet [21]
resnet/res50 640 × 640	ResNet [21]
resnet/res101 512 × 512	ResNet [21]
resnet/res152 512 × 512	ResNet [21]
hrnet w32 512 × 512 udp	HRNet [30] UDP [28]
higher hrnet32 512 × 512 udp	Higher HRNet [31] UDP [28]

**Table 3:** Comparison of selected methods for test1. Methods are sorted by increasing Average Precision AP${}_{y}^{50}$. Therefore, the best (most accurate) methods appear at the bottom of the table. The prefix of each method indicates its type: DT = detection, MK = mask-based, BU = bottom-up pose detection, TD = top-down pose detection. See Table 1 and Table 2 for abbreviations on top-down and bottom-up methods, respectively.

Method	$\bar{|E_{x}|}$	$s(E_{x})$	AP${}_{x}^{10}$	AP${}_{x}^{20}$	$\bar{|E_{y}|}$	$s(E_{y})$	AP${}_{y}^{30}$	AP${}_{y}^{40}$	AP${}_{y}^{50}$
DT-htc-x101-64 × 4d	10.87	13.48	54.3	86.1	59.41	18.71	0.3	0.3	0.6
DT-faster-rcnn-r50-1x	10.83	13.49	55.6	87.0	58.19	21.48	0.7	1.0	1.2
MK-Detectron2	9.25	12.67	66.9	87.7	40.86	23.51	3.0	3.8	5.5
BU-openpose	4.03	5.50	92.3	99.1	22.59	23.85	40.0	63.3	73.6
BU-mobilenetv2-512 × 512	4.62	8.93	91.4	98.6	21.59	25.38	58.1	75.7	85.5
BU-res50-640 × 640	3.73	6.33	95.1	98.9	19.41	20.48	52.8	76.7	87.5
BU-hrnet-w32-512 × 512	3.06	4.54	97.9	99.8	17.38	15.98	51.7	75.9	88.6
BU-res152-512 × 512	3.14	4.14	97.7	99.8	17.67	18.74	62.3	81.1	88.9
BU-higher-hrnet32-512 × 512	2.55	3.29	98.9	100.0	16.48	13.87	53.1	78.0	91.2
BU-higher-hrnet32-640 × 640	2.68	3.45	98.7	100.0	15.60	13.29	60.6	83.3	94.1
TD-faster-rcnn-r50-1x—resnetv1d152-384 × 288	2.80	3.45	98.6	99.9	15.41	12.76	59.5	85.1	94.9
TD-faster-rcnn-r50-1x—hourglass52-384 × 384	2.82	3.54	98.0	99.9	15.00	12.41	61.4	87.9	95.3
TD-faster-rcnn-r50-1x—hrnet-w48-384 × 288-dark	2.78	4.11	98.1	99.8	14.29	11.80	67.4	88.4	95.9
TD-faster-rcnn-r50-1x—hrnet-w48-384 × 288	2.68	3.26	98.7	100.0	14.40	11.55	65.2	89.9	97.0
TD-cascade-mask-rcnn-x101-64 × 4d—hrnet-w48-256 × 192	2.67	3.37	98.1	99.9	14.16	11.42	67.9	90.0	97.2
TD-cascade-mask-rcnn-x101-32 × 4d—hrnet-w48-256x192	2.66	3.39	98.2	99.8	14.27	11.42	66.5	89.0	97.2
TD-htc-x101-64 × 4d—hrnet-w48-256 × 192	2.70	3.37	98.5	99.9	14.20	11.30	67.5	89.7	97.5
TD-faster-rcnn-r50-1x—hrnet-w48-256 × 192-person	2.75	4.23	98.3	99.7	11.12	10.93	87.6	96.9	98.2
TD-faster-rcnn-x101-64 × 4d-1x—hrnet-w48-256 × 192	2.63	3.23	98.5	100.0	11.10	10.66	88.0	96.9	98.7
TD-faster-rcnn-r50-1x—hrnet-w48-256 × 192	2.71	4.13	98.4	99.9	11.34	11.47	87.3	95.9	98.7

**Table 4:** Comparison of selected methods for test1, for players *on the bottom half of the court*. Methods are sorted by increasing Average Precision AP${}_{y}^{50}$. Therefore, the best (most accurate) methods appear at the bottom of the table. The prefix of each method indicates its type: DT = detection, MK = mask-based, BU = bottom-up pose detection, TD = top-down pose detection. See Table 1 and Table 2 for abbreviations on top-down and bottom-up methods, respectively.

Method	$\bar{|E_{x}|}$	$s(E_{x})$	AP${}_{x}^{10}$	AP${}_{x}^{20}$	$\bar{|E_{y}|}$	$s(E_{y})$	AP${}_{y}^{30}$	AP${}_{y}^{40}$	AP${}_{y}^{50}$
DT-htc-x101-64 × 4d	11.67	14.21	49.2	84.1	51.73	12.87	0.0	0.0	0.2
DT-faster-rcnn-r50-1x	11.77	14.35	48.4	84.5	50.29	13.37	0.0	0.0	0.2
MK-Detectron2	10.13	13.62	63.1	83.8	33.43	16.23	1.9	2.6	6.2
BU-mobilenetv2-512 × 512	4.02	9.99	96.8	98.2	17.66	22.44	72.3	89.7	93.1
BU-res50-640 × 640	3.22	5.71	97.4	99.3	14.69	17.00	83.5	93.5	97.1
BU-res152-512 × 512	2.70	3.36	99.0	100.0	12.36	10.39	83.6	96.0	99.0
TD-faster-rcnn-r50-1x—hrnet-w48-256 × 192	2.57	4.53	99.0	99.8	7.31	7.30	98.7	99.6	99.6
BU-openpose	3.04	3.84	98.1	100.0	10.53	11.95	95.4	99.2	99.6
BU-hrnet-w32-512 × 512	2.57	3.14	99.6	100.0	10.56	8.91	91.9	99.0	99.6
BU-higher-hrnet32-512 × 512	2.36	2.87	99.8	100.0	10.03	7.72	94.7	99.5	99.8
TD-cascade-mask-rcnn-x101-32 × 4d—hrnet-w48-256 × 192	2.47	2.91	99.2	100.0	9.31	7.21	97.5	99.8	99.8
TD-faster-rcnn-r50-1x—hrnet-w48-384 × 288	2.55	2.94	98.8	100.0	9.67	7.29	96.2	99.8	99.8
TD-faster-rcnn-r50-1x—hrnet-w48-384 × 288-dark	2.64	4.37	98.5	99.8	8.86	7.12	98.1	99.6	99.8
TD-faster-rcnn-r50-1x—resnetv1d152-384 × 288	2.58	2.99	99.2	100.0	9.47	7.66	97.3	99.6	99.8
TD-cascade-mask-rcnn-x101-64 × 4d—-hrnet-w48-256 × 192	2.53	3.00	99.0	100.0	9.07	6.82	98.3	100.0	100.0
BU-higher-hrnet32-640 × 640	2.46	3.08	99.0	100.0	9.45	7.60	97.7	100.0	100.0
TD-faster-rcnn-r50-1x—hourglass52-384 × 384	2.66	3.12	98.3	100.0	9.74	7.55	96.0	99.6	100.0
TD-faster-rcnn-r50-1x—hrnet-w48-256 × 192-person	2.51	3.88	99.0	99.8	7.11	6.67	98.8	99.8	100.0
TD-faster-rcnn-x101-64 × 4d-1x—hrnet-w48-256 × 192	2.43	2.84	99.0	100.0	7.10	6.52	99.0	100.0	100.0
TD-htc-x101-64 × 4d—hrnet-w48-256 × 192	2.54	3.01	99.2	100.0	9.22	6.88	98.1	99.8	100.0

## Data Availability

The test images for the study were collected from publicly available videos. Links to these videos are included in Table S1 of the Supplemental Material. The computer vision methods used to analyze the test images are available in OpenMMLab https://github.com/open-mmlab, (accessed on 1 February 2021) and OpenPose https://github.com/CMU-Perceptual-Computing-Lab/openpose, (accessed on 1 February 2021) repositories.

## Supplementary Materials

The following are available online at https://www.mdpi.com/article/10.3390/s21103368/s1, Table S1: WPT padel matches selected for the evaluation, Video S1: Estimated player positions for the amateur padel video.

## References

[1] Santiago C.B., Sousa A., Estriga M.L., Reis L.P., Lames M. Survey on team tracking techniques applied to sports. Proceedings of the 2010 International Conference on Autonomous and Intelligent Systems.

[2] Shih H.C. (2017). A survey of content-aware video analysis for sports. IEEE Trans. Circuits Syst. Video Technol..

[3] Priego J.I., Melis J.O., Belloch S.L., Soriano P.P., García J.C.G., Almenara M.S. (2013). Padel: A Quantitative study of the shots and movements in the high-performance. J. Hum. Sport Exerc..

[4] Yazaki S., Yamamoto O. (2007). Analyzing Movements of Tennis Players by Dynamic Image Processing. IEEJ Trans. Electron. Inf. Syst..

[5] Mukai R., Araki T., Asano T. (2013). Quantitative Evaluation of Tennis Plays by Computer Vision. IEEJ Trans. Electron. Inf. Syst..

[6] Lara J.P.R., Vieira C.L.R., Misuta M.S., Moura F.A., Barros R.M.L.D. (2018). Validation of a video-based system for automatic tracking of tennis players. Int. J. Perform. Anal. Sport.

[7] Pingali G., Opalach A., Jean Y. Ball tracking and virtual replays for innovative tennis broadcasts. Proceedings of the 15th International Conference on Pattern Recognition, ICPR-2000.

[8] Mao J. (2006). Tracking a Tennis Ball Using Image Processing Techniques. Ph.D. Thesis.

[9] Qazi T., Mukherjee P., Srivastava S., Lall B., Chauhan N.R. Automated ball tracking in tennis videos. Proceedings of the 2015 Third International Conference on Image Information Processing (ICIIP).

[10] Kamble P.R., Keskar A.G., Bhurchandi K.M. (2019). Ball tracking in sports: A survey. Artif. Intell. Rev..

[11] Sudhir G., Lee J.C.M., Jain A.K. Automatic classification of tennis video for high-level content-based retrieval. Proceedings of the 1998 IEEE International Workshop on Content-Based Access of Image and Video Database.

[12] Dahyot R., Kokaram A., Rea N., Denman H. Joint audio visual retrieval for tennis broadcasts. Proceedings of the 2003 IEEE International Conference on Acoustics, Speech, and Signal Processing.

[13] Yan F., Christmas W., Kittler J. A tennis ball tracking algorithm for automatic annotation of tennis match. Proceedings of the British Machine Vision Conference.

[14] Ramón-Llin J., Guzmán J., Martínez-Gallego R., Muñoz D., Sánchez-Pay A., Sánchez-Alcaraz B.J. (2020). Stroke Analysis in Padel According to Match Outcome and Game Side on Court. Int. J. Environ. Res. Public Health.

[15] Mas J.R.L., Belloch S.L., Guzmán J., Vuckovic G., Muñoz D., Martínez B.J.S.A. (2020). Análisis de la distancia recorrida en pádel en función de los diferentes roles estratégicos y el nivel de juego de los jugadores (Analysis of distance covered in padel based on level of play and number of points per match). Acción Motriz.

[16] Vučković G., Perš J., James N., Hughes M. (2010). Measurement error associated with the SAGIT/Squash computer tracking software. Eur. J. Sport Sci..

[17] Ramón-Llin J., Guzmán J.F., Llana S., Martínez-Gallego R., James N., Vučković G. (2019). The Effect of the Return of Serve on the Server Pair’s Movement Parameters and Rally Outcome in Padel Using Cluster Analysis. Front. Psychol..

[18] Baclig M.M., Ergezinger N., Mei Q., Gül M., Adeeb S., Westover L. (2020). A Deep Learning and Computer Vision Based Multi-Player Tracker for Squash. Appl. Sci..

[19] Cao Z., Hidalgo G., Simon T., Wei S.E., Sheikh Y. (2019). OpenPose: Realtime multi-person 2D pose estimation using Part Affinity Fields. IEEE Trans. Pattern Anal. Mach. Intell..

[20] Chen K., Wang J., Pang J., Cao Y., Xiong Y., Li X., Sun S., Feng W., Liu Z., Xu J. (2019). MMDetection: Open MMLab Detection Toolbox and Benchmark. arXiv.

[21] Xiao B., Wu H., Wei Y. Simple baselines for human pose estimation and tracking. Proceedings of the European Conference on Computer Vision (ECCV).

[22] Ren S., He K., Girshick R., Sun J. (2017). Faster R-CNN: Towards Real-Time Object Detection with Region Proposal Networks. IEEE Trans. Pattern Anal. Mach. Intell..

[23] Cai Z., Vasconcelos N. (2019). Cascade R-CNN: High Quality Object Detection and Instance Segmentation. IEEE Trans. Pattern Anal. Mach. Intell..

[24] Wu Y., Kirillov A., Massa F., Lo W.Y., Girshick R. (2019). Detectron2. https://github.com/facebookresearch/detectron2.

[25] Chen K., Pang J., Wang J., Xiong Y., Li X., Sun S., Feng W., Liu Z., Shi J., Ouyang W. Hybrid task cascade for instance segmentation. Proceedings of the IEEE Conference on Computer Vision and Pattern Recognition.

[26] Sandler M., Howard A., Zhu M., Zhmoginov A., Chen L.C. Mobilenetv2: Inverted residuals and linear bottlenecks. Proceedings of the IEEE Conference on Computer Vision and Pattern Recognition.

[27] Newell A., Yang K., Deng J. Stacked hourglass networks for human pose estimation. Proceedings of the European Conference on Computer Vision.

[28] Huang J., Zhu Z., Guo F., Huang G. The Devil Is in the Details: Delving Into Unbiased Data Processing for Human Pose Estimation. Proceedings of the IEEE/CVF Conference on Computer Vision and Pattern Recognition (CVPR).

[29] Zhang F., Zhu X., Dai H., Ye M., Zhu C. Distribution-aware coordinate representation for human pose estimation. Proceedings of the IEEE/CVF Conference on Computer Vision and Pattern Recognition.

[30] Sun K., Xiao B., Liu D., Wang J. Deep high-resolution representation learning for human pose estimation. Proceedings of the IEEE Conference on Computer Vision and Pattern Recognition.

[31] Cheng B., Xiao B., Wang J., Shi H., Huang T.S., Zhang L. HigherHRNet: Scale-Aware Representation Learning for Bottom-Up Human Pose Estimation. Proceedings of the IEEE/CVF Conference on Computer Vision and Pattern Recognition.

[32] Zhao Z.Q., Zheng P., Xu S.T., Wu X. (2019). Object detection with deep learning: A review. IEEE Trans. Neural Netw. Learn. Syst..

[33] Loper M., Mahmood N., Romero J., Pons-Moll G., Black M.J. (2015). SMPL: A Skinned Multi-Person Linear Model. ACM Trans. Graphics (Proc. SIGGRAPH Asia).

[34] Raykar V.C., Yu S., Zhao L.H., Valadez G.H., Florin C., Bogoni L., Moy L. (2010). Learning from crowds. J. Mach. Learn. Res..

[35] He K., Zhang X., Ren S., Sun J. Deep residual learning for image recognition. Proceedings of the IEEE Conference on Computer Vision and Pattern Recognition.

[36] Xie S., Girshick R., Dollár P., Tu Z., He K. Aggregated residual transformations for deep neural networks. Proceedings of the IEEE Conference on Computer Vision and Pattern Recognition.

[37] Zhu X., Hu H., Lin S., Dai J. (2018). Deformable ConvNets v2: More Deformable, Better Results. arXiv.

[38] Skublewska-Paszkowska M., Powroznik P., Lukasik E. (2020). Learning Three Dimensional Tennis Shots Using Graph Convolutional Networks. Sensors.

[39] Steels T., Van Herbruggen B., Fontaine J., De Pessemier T., Plets D., De Poorter E. (2020). Badminton Activity Recognition Using Accelerometer Data. Sensors.

[40] Yu C.H., Wu C.C., Wang J.S., Chen H.Y., Lin Y.T. (2020). Learning tennis through video-based reflective learning by using motion-tracking sensors. J. Educ. Technol. Soc..

[41] Delgado-García G., Vanrenterghem J., Ruiz-Malagón E.J., Molina-García P., Courel-Ibáñez J., Soto-Hermoso V.M. (2020). IMU gyroscopes are a valid alternative to 3D optical motion capture system for angular kinematics analysis in tennis. Proc. Inst. Mech. Eng. Part J. Sport. Eng. Technol..

[42] Zhu X., Xiong Y., Dai J., Yuan L., Wei Y. Deep feature flow for video recognition. Proceedings of the IEEE Conference on Computer Vision and Pattern Recognition.

[43] Zhu X., Wang Y., Dai J., Yuan L., Wei Y. Flow-guided feature aggregation for video object detection. Proceedings of the IEEE International Conference on Computer Vision.

[44] Wu H., Chen Y., Wang N., Zhang Z. Sequence level semantics aggregation for video object detection. Proceedings of the IEEE International Conference on Computer Vision.

[45] Wojke N., Bewley A., Paulus D. Simple online and realtime tracking with a deep association metric. Proceedings of the 2017 IEEE international conference on image processing (ICIP).

[46] Bergmann P., Meinhardt T., Leal-Taixe L. Tracking without bells and whistles. Proceedings of the IEEE International Conference on Computer Vision.

[47] Uzu R., Shinya M., Oda S. (2009). A split-step shortens the time to perform a choice reaction step-and-reach movement in a simulated tennis task. J. Sport. Sci..

[48] Pons E., Ponce-Bordón J.C., Díaz-García J., López del Campo R., Resta R., Peirau X., García-Calvo T. (2021). A Longitudinal Exploration of Match Running Performance during a Football Match in the Spanish La Liga: A Four-Season Study. Int. J. Environ. Res. Public Health.

[49] Cust E.E., Sweeting A.J., Ball K., Robertson S. (2019). Machine and deep learning for sport-specific movement recognition: A systematic review of model development and performance. J. Sport. Sci..

[50] Passos P., Lacasa E., Milho J., Torrents C. (2020). Capturing Interpersonal Synergies in Social Settings: An Example within a Badminton Cooperative Task. Nonlinear Dyn. Psychol. Life Sci..

[51] Linke D., Link D., Lames M. (2018). Validation of electronic performance and tracking systems EPTS under field conditions. PLoS ONE.

[52] Yan S., Xiong Y., Lin D. Spatial temporal graph convolutional networks for skeleton-based action recognition. Proceedings of the AAAI Conference on Artificial Intelligence.

