# Supplementary material for: Estimating Player Positions from Padel High-Angle Videos: Accuracy Comparison of Recent Computer Vision Methods
Source: Sensors (Basel). 2021 May 12;21(10):3368. doi: 10.3390/s21103368 (PMC8151013; doi:10.3390/s21103368)
Supplement: Supplementary file 1 [file sensors-21-03368-s001.zip › sensors-1170080-supplementary-final/supplementary-table/template.pdf]

sensors

MDPI

Article

Estimating Player Positions from Padel High-angle Videos: Accuracy Comparison of Recent Computer Vision Methods

Mohammadreza Javadiha [1,†], Carlos Andujar [2,*,†], Enrique Lacasa [3], Angel Ric [3] and Antonio Susin [4]

1 ViRVIG, Universitat Politècnica de Catalunya-BarcelonaTech; Pau Gargallo 14, CS Dept, Edifici U, Barcelona 08028, Spain; mohammadreza.javadiha@upc.edu
2 ViRVIG, Universitat Politècnica de Catalunya-BarcelonaTech; Jordi Girona 1-3, CS Dept, Edifici Omega, Barcelona 08034, Spain; andujar@cs.upc.edu
3 Complex Systems in Sport Research Group, Institut Nacional D'Educacio Fisica de Catalunya (INEFC), University of Lleida (UdL), Lleida 25192, Spain; elacasa@inefc.es (E.L.); aric@gencat.cat (A.R.)
4 ViRVIG-Universitat Politècnica de Catalunya-BarcelonaTech; Avda. Diagonal 647, Engineering School (ETSEIB), Barcelona 08028, Spain; toni.susin@upc.edu

check for updates

Citation: Javadiha, M.; Andujar, C.; Lacasa, E.; Ric, A; Susin, A. Estimating Player Positions from Padel High-angle Videos: Accuracy Comparison of Recent Computer Vision Methods. *Sensors* **2021**, *1*, 0. https://doi.org/

Received:
Accepted:
Published:

Publisher's Note: MDPI stays neutral with regard to jurisdictional claims in published maps and institutional affiliations.

Copyright: © 2020 by the authors. Licensee MDPI, Basel, Switzerland. This article is an open access article distributed under the terms and conditions of the Creative Commons Attribution (CC BY) license (https://creativecommons.org/licenses/by/4.0/).

1. Supplemental material - Dataset description

Our evaluation is based on frames selected from professional padel videos. In particular, we selected 24 matches from World Pader Tour, all of them publicly available on the WPT YouTube channel (Table S1). The selected set is varied in terms of gender (15 male, 9 female finals) and lighting conditions (16 indoor, 8 outdoor).

Table 1. WPT padel matches selected for the evaluation.

Tournament	Match	City	Length	Light	URL
Buenos Aires Padel Master 2019	Male final	Buenos Aires	00:40:37	Indoor	Link
Estrella DAMM Master FINAL 2017	Female final	Madrid	01:55:26	Indoor	Link
Estrella DAMM Master FINAL 2017	Male final	Madrid	01:21:35	Indoor	Link
Euro Finans Swedish Open 2018	Male final	Bastad	02:04:45	Outdoor	Link
Santander WOpen 2019	Female final	Santander	02:23:50	Indoor	Link
Buenos Aires Padel Master 2017	Male final	Buenos Aires	01:40:05	Outdoor	Link
Estrella Damm Alicante Open 2017	Female final	Alicante	01:39:05	Indoor	Link
Estrella Damm Alicante Open 2017	Male final	Alicante	01:58:10	Indoor	Link
Estrella Damm Zaragoza Open 2017	Female final	Zaragoza	02:32:40	Indoor	Link
Estrella Damm Zaragoza Open 2017	Male final	Zaragoza	01:11:15	Indoor	Link
Cerveza Victoria Mijas - Costa del Sol Open 2017	Male final	Mijas	02:45:10	Outdoor	Link
Portugal Padel Master 2017	Male final	Lisbon	01:37:40	Outdoor	Link
Santander Open 2017	Male final	Santander	02:27:53	Indoor	Link
Santander Open 2017	Female final	Santander	02:06:48	Indoor	Link
Sevilla Open 2017	Female final	Sevilla	01:05:17	Outdoor	Link
Sevilla Open 2017	Male final	Sevilla	01:07:52	Outdoor	Link
Estrella Damm Madrid Master 2019	Female final	Madrid	01:29:58	Indoor	Link
Estrella Damm Madrid Master 2019	Male final	Madrid	01:36:19	Indoor	Link
Bilbao Open 2018	Male final	Bilbao	01:19:50	Indoor	Link
Bilbao Open 2018	Female final	Bilbao	02:31:14	Indoor	Link
Buenos Aires Padel Master 2018	Male final	Buenos Aires	02:21:52	Outdoor	Link
Cascais Padel Master 2019	Male final	Cascais	00:26:54	Outdoor	Link
Cervezas Victoria Córdoba Open 2019	Female final	Córdoba	01:34:21	Indoor	Link
Cervezas Victoria Córdoba Open 2019	Male final	Córdoba	01:12:09	Indoor	Link
