# Supplementary figures and images for: Estimating Player Positions from Padel High-Angle Videos: Accuracy Comparison of Recent Computer Vision Methods
Source: Sensors (Basel). 2021 May 12;21(10):3368. doi: 10.3390/s21103368 (PMC8151013; doi:10.3390/s21103368)

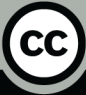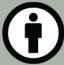

BY

Supplement: Supplementary file 1 [file sensors-21-03368-s001.zip › sensors-1170080-supplementary-final/supplementary-table/Definitions/logo-ccby-eps-converted-to.pdf]

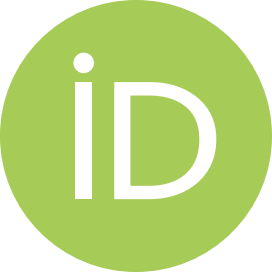

Supplement: Supplementary file 1 [file sensors-21-03368-s001.zip › sensors-1170080-supplementary-final/supplementary-table/Definitions/logo-orcid-eps-converted-to.pdf]

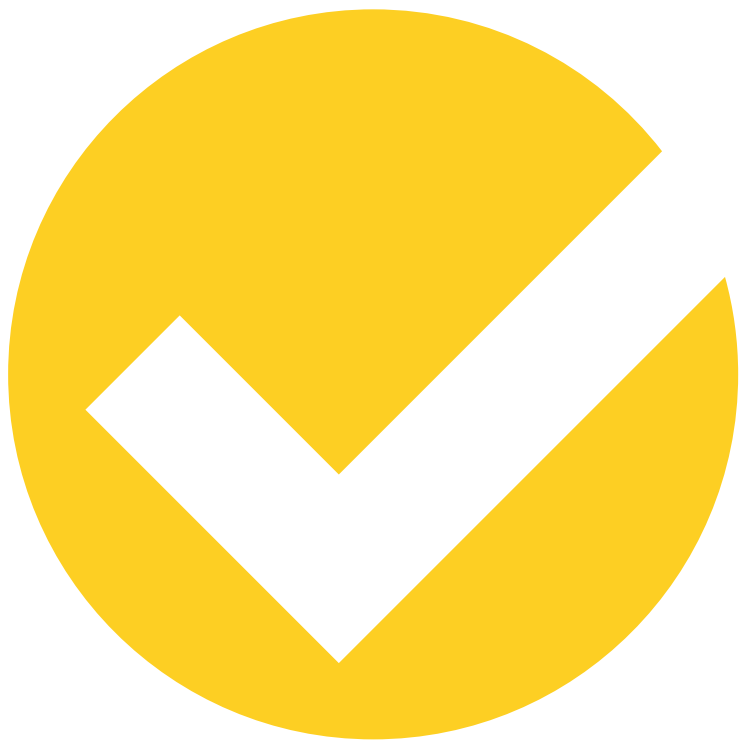

check for  
updates

Supplement: Supplementary file 1 [file sensors-21-03368-s001.zip › sensors-1170080-supplementary-final/supplementary-table/Definitions/logo-updates.pdf]

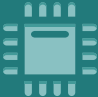

*sensors*

Supplement: Supplementary file 1 [file sensors-21-03368-s001.zip › sensors-1170080-supplementary-final/supplementary-table/Definitions/sensors-logo-eps-converted-to.pdf]

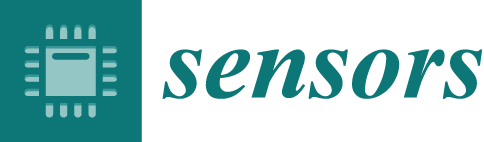

Supplement: Supplementary file 1 [file sensors-21-03368-s001.zip › sensors-1170080-supplementary-final/supplementary-table/Definitions/sensors-logo.png]
